# Improving Biomethane Production and Mass Bioconversion of Corn Stover Anaerobic Digestion by Adding NaOH Pretreatment and Trace Elements

**DOI:** 10.1155/2015/125241

**Published:** 2015-06-02

**Authors:** ChunMei Liu, HaiRong Yuan, DeXun Zou, YanPing Liu, BaoNing Zhu, XiuJin Li

**Affiliations:** College of Chemical Technology, Centre for Resource and Environmental Research, Beijing University of Chemical Technology, 15 Beisanhuan East Road, Chaoyang District, Beijing 100029, China

## Abstract

This research applied sodium hydroxide (NaOH) pretreatment and trace elements to improve biomethane production when using corn stover for anaerobic digestion. Full-factor experimental tests identified the best combination of trace elements with the NaOH pretreatment, indicating that the best combination was with 1.0, 0.4, and 0.4 mg·L^−1^·d^−1^ of elements Fe, Co, and Ni, respectively. The cumulative biomethane production adding NaOH pretreatment and trace elements was 11,367 mL; total solid bioconversion rate was 55.7%, which was 41.8%–62.2% higher than with NaOH-pretreatment alone and 22.2%–56.3% higher than with untreated corn stover. The best combination was obtained 5–9 days shorter than T_90_ and maintained good system operation stability. Only a fraction of the trace elements in the best combination was present in the resulting solution; more than 85% of the total amounts added were transferred into the solid fraction. Adding 0.897 g of Fe, 0.389 g of Co, and 0.349 g of Ni satisfied anaerobic digestion needs and enhanced biological activity at the beginning of the operation. The results showed that NaOH pretreatment and adding trace elements improve corn stover biodegradability and enhance biomethane production.

## 1. Introduction

Corn is one of the three major crops in China and is widely planted in the northern part of China. Corn stover is one of the most abundant lignocellulosic crop residues, with an annual production of 0.1 billion tons. Most of this residue remains unused [1]. It is quite common to see the open-field burning of corn stover across corn planting areas during the harvest season; these fires lead to serious air pollution and fire disaster and threaten traffic safety.

Biomethane production through anaerobic digestion is an energy-efficient and environmentally friendly way to treat and reuse agricultural organic materials. These wastes can be used as alternative feedstock to produce renewable energy, biomethane, valuable digested residues, liquid fertilizers, and soil conditioners [2]. However, anaerobic digestion is not currently popular, because of its poor biodegradability and digestibility. This is particularly true with treating crop residues.

Corn stover is mainly composed of polysaccharide (cellulose and hemicellulose) and lignin, forming complex three-dimensional structures. The native cellulose fraction of corn stover resists enzymatic breakdown due to the complex structure of lignin and hemicellulose with the cellulose, making enzymatic disassembly difficult [3]. To obtain fast enzymatic hydrolysis of feedstock with a high sugar yield, the cell structures must be broken and porosity increased. Therefore, pretreatment is required to prepare the native cellulose fraction for enzymatic hydrolysis to monosaccharides.

Generally, pretreatment methods are classified into physical pretreatments (i.e., milling, liquid hot water, and steam), chemical pretreatments (alkaline, acidic, and oxidative), and biological pretreatments (i.e., commercial enzymes or fungi). Previous studies have found that alkali pretreatment is the best known method for enhancing complex material biodegradation and providing the most significant benefits [4].

In addition to pretreatment challenges, existing trace elements and nitrogen are insufficient for anaerobic microorganisms when corn stover is used alone as a feedstock for anaerobic digestion. This results in a decrease in biogas production after a certain treatment period and a process failure if no external nutrients and buffering agents are added [5]. Misunderstanding or underestimating trace nutrient requirements of methanogens can also be a serious problem in applying anaerobic biotechnology, and trace element availability as micronutrients plays a significant role in the performance and stability of substrates ranging from organic household waste to more defined lignocellulosic substrates [6].

Trace elements such as cobalt, nickel, iron, tungsten, or molybdenum serve as enzyme cofactors and are involved in the biomethane formation biochemistry [7]. Li and Dong (2001) reported that the most required trace elements were Fe, Co, and Ni [8]. For example, methyl-coenzyme M and cofactor F_430_ contain nickel; the acetate converting enzyme complex carbon monoxide dehydrogenase (CODH) contains a nickel-iron-sulfur component; and the methyl-H_4_SPT contains cobalt [9]. However, no information was found on the influence of trace elements on biomethane production and mass bioconversion of corn stover sole substrate.

Elemental deficiencies may negatively influence biological processes and biomethane formation; on the contrary, higher concentration of trace elements may be toxic to methanogens [10]. Consequently, adequate trace element concentrations (Fe, Co, and Ni) must be quantified accurately when added to an experimental reactor. However, essential trace element availability for the bacterial community is still a concern when studying single substrates rather than complex material mixtures, as different forms of trace elements have not been studied.

This study's objective was to determine the optimal combination of the trace elements Fe, Co, and Ni and to investigate the performance and synergistic effect of combining NaOH pretreatment with additional trace elements during corn stover anaerobic digestion. Factors evaluated included biogas production, mass bioconversion, and trace element bioavailability.

## 2. Materials and Methods

### 2.1. Feedstock and Inoculum

The corn stover used in this study was collected from Beichen County of Tianjin City, China. The corn stover was chopped using a paper chopper (PC500, Staida Co., Tianjin, China) and then ground through a 20 mesh screen using a universal pulverizer (YSW-180, Yanshan Zhengde Co., Beijing, China). Previous study revealed that a NaOH dose of 2%, 88% moisture content, and a 3-day treatment time were appropriate for wet state NaOH pretreatment of corn stover [1]. This method was selected because of the improvement in corn stover's biodegradability and environmental friendliness. The wet state NaOH pretreatment was conducted in a laboratory at ambient temperature (20 ± 2°C) for three days.

The sludge (Inoculum) was collected from Shunyi County of Beijing City, China.  Table 1 provides corn stover and sludge characteristics.

### 2.2. Experimental Set-Up

NaOH-pretreated corn stover was added with the three trace elements in batch anaerobic digesters, to investigate the combined effect of the trace elements and NaOH pretreatment on digestion performance.

Different concentrations of a well-defined trace element solution were added to anaerobic batch experiments according to our research and other authors' research [11]. To study the interaction of elements Fe (FeCl_2_·4H_2_O), Co (CoCl_2_·6H_2_O), and Ni (NiCl_2_·6H_2_O), a full-factor test was designed to find the best element combinations; Table 2 shows the coding factors and levels. Higher trace element concentrations had inhibitory and toxic effects on anaerobic digestion [12]. As such, it was necessary to determine the trace metal amounts required to supplement levels already present in the substrate and measure levels in the digester's solid and liquid residue streams to assess potential toxicity issues in their use and disposal.

Digestion experiments were performed in batch anaerobic digesters, and each experiment was repeated three times. Each digester was 1 L in volume, with a working volume of 0.8 L. A loading rate of 65 g·TS/L was applied for the NaOH-pretreated corn stover. Each digester was seeded to maintain the sludge MLSS (mixed liquid suspended solids) in the digester at 15 g/L [13]. Urea was added to each digester to adjust the carbon-to-nitrogen ratio (C/N) to 25, believed to be optimal for anaerobic bacteria growth. The pH was adjusted to 7.5 ± 0.1 using calcium hydroxide (Ca(OH)_2_) solution at the beginning of the anaerobic digestion process. Prepared digesters were then placed in a water bath for anaerobic digestion tests. The water bath was operated at mesophilic temperature (35 ± 1°C) for a time period of 50 days.

Batch experiments were conducted for the best combination. Untreated (raw) and NaOH-pretreated corn stover were used as separate control samples, with the same operating conditions described above.

### 2.3. Analytical Methods

#### 2.3.1. Biogas Analyses

Each anaerobic digester's biogas production was recorded daily using the water displacement method, and the corresponding cumulative biogas volume was calculated. The measured volume was then converted to a biogas volume at a standard temperature and pressure using the ideal gas law; this volume was used to calculate biomethane volume based on the BVF (biomethane volume fraction). The biogas BVF was analyzed daily using a gas chromatograph (GC) (SP-2100, BeiFenRuiLi Co., Beijing, China) equipped with a molecular sieve (TDX-01) packed 2 m × 3 mm stainless-steel column and a thermal conductivity detector (TCD). The temperatures of the oven, injector port, and TCD were 140, 150, and 150°C, respectively. Argon was used as the carrier gas at a 30 mL min^−1^ flow rate. A standard gas (BeiFenRuiLi Co., Beijing), composed of 10.02% H_2_, 4.98% N_2_, 50.1% CH_4_, and 34.9% CO_2_, was used to calibrate the system.

#### 2.3.2. Chemical Composition Analyses

Total solids (TS), volatile solids (VS), and mixed liquor suspended solids (MLSS) of the corn stover, sludge, and their mixture were measured using APHA standard methods. Total carbon (TC) and total nitrogen (TN) were determined using a Vario EL/microcube elemental analyzer (Elementar, Germany). Lignin, cellulose, and hemicellulose content were determined using an automatic fiber analyzer (ANKOM A2000i, ANKOM, USA) using procedures proposed by Van Soest [14]. The pH was measured with a pH meter (3-Star, Thermo Orion, USA). Trace elements were quantified using an inductively coupled plasma optical emission spectrometer (iCAP 7500, Thermo Scientific, USA).

### 2.4. Data Analyses

Each analytical datum was the mean of at least three measurements. Full-factor test results, the standard deviations, and analysis of variance were analyzed using the statistical software SPSS 17.0 for Windows; analysis of variance was tested using a least-significant difference (LSD) method.

## 3. Results and Discussion

### 3.1. Determination of Optimal Trace Element Level

Serial batch experiments were conducted to investigate the effect of adding trace elements on biomethane production and to determine optimal trace element levels. All corn stover samples used for trace element tests were pretreated with NaOH.  Table 3 presents biomethane productions with different concentrations of Fe, Co, and Ni. Batch trials with the combination A1B3C2 achieved the highest biomethane production, with concentrations of Fe, Co, and Ni at 1.0 mg·L^−1^·d^−1^, 0.4 mg·L^−1^·d^−1^, and 0.4 mg·L^−1^·d^−1^, respectively. The result is similar to findings from Li and Dong (2001), who reported an increase of biogas production after adding the combination of Fe (0.3 mg·L^−1^·d^−1^), Co (0.05 mg·L^−1^·d^−1^), and Ni (0.20 mg·L^−1^·d^−1^) during anaerobic digestion [8].

Table 4 shows between-subject effects. Factor B and the interaction of A∗B∗C, B∗C, A∗C were highly significant. Factors A and C and the interaction of A∗B had no effect on the test results. In terms of their respective influence, A∗B∗C was greater than B, which was greater than B∗C, which was greater than A∗C. The three trace elements Fe, Co, and Ni interacted with one another; the interaction of all three trace elements was higher than the interaction of a single element and two elements. Speece noted that the interaction among Fe, Co, Ni, Mo, and Se plays an important role in anaerobic digestion processes [6]. As such, group A1B3C2 was considered to be the optimal trace element combination and was used for subsequent tests.

### 3.2. Biomethane Production

#### 3.2.1. Daily Biogas and Biomethane Production

**Figure 1:**
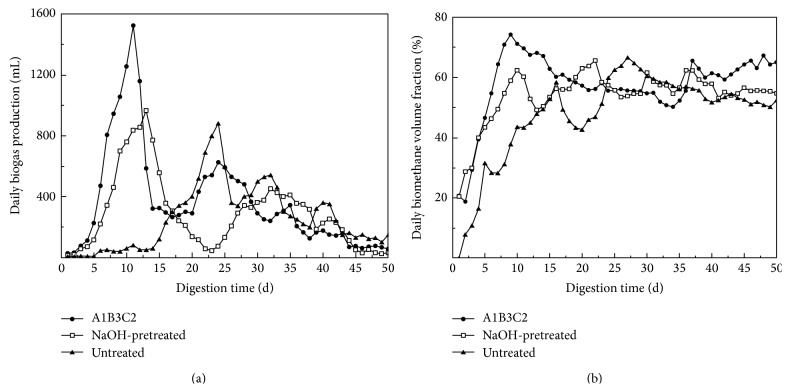
The daily biogas production (a) and biomethane volume fraction (b) for different groups.

The daily biogas production and the biomethane volume fraction for each group were recorded throughout the digestion test period.  Figure 1(a) shows the daily biogas productions for different groups, demonstrating that the overall change trends were very similar. All daily biogas productions experienced fluctuation; biogas generation started after seeding and experienced several small peaks before finally ceasing. The start-up time, the biogas production peak value, and the time of the peak value which was reached differed for different groups. The digesters with A1B3C2 and NaOH-pretreated corn stover experienced rapid start-up after seeding. The highest daily biogas production reached 1,525 mL on Day 11 for the A1B3C2 group. For the NaOH-pretreated group, production reached 965 mL on Day 13; for the untreated group, production reached 880 mL on Day 24. Compared to the untreated corn stover and NaOH-pretreated corn stover, results indicate that the A1B3C2 group reached higher daily biogas production within a shorter digestion time.

Figure 1(b) shows the biomethane volume fraction (BVF) of the three groups. All showed similar general trends, with the BVF first increasing and then levelling off at a relatively constant level. However, the BVFs of the A1B3C2 and NaOH-pretreated groups increased quickly at start-up and reached constant levels earlier. The BVF of the untreated group increased slowly and took a longer time to reach a constant level. The average BVF of the A1B3C2 group was 61.8%; this is higher than the BVF of 55.7% for the NaOH-pretreated group and the BVF of 50.8% for the untreated group.

This finding indicates that combining NaOH pretreatment with supplemental trace elements could increase BVF. The result agrees with a study by Chen et al., who reported that the biogas volume increased by 43.4% and that BVF increased by 5.1% when Fe, Co, and Ni were applied at concentrations of 1.0, 0.1, and 0.2 mg·L^−1^·d^−1^, respectively [15]. A higher BVF may bring significant economic benefit, as it increases the biomethane yield for a given amount of corn stover.

#### 3.2.2. Cumulative Biogas and Biomethane Production

**Figure 2:**
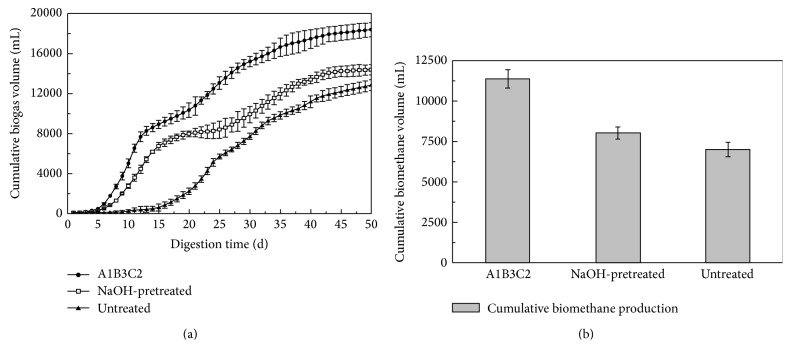
Cumulative biogas production (a) and biomethane production (b) for different groups.

Cumulative biogas production was calculated based on the daily biogas production during anaerobic fermentation.  Figure 2(a) shows the changes in cumulative biogas productions for the three groups studied. After 50 days of digestion, the A1B3C2 group reached the highest cumulative biogas production of 18,400 mL. This was 27.9% higher than tests with NaOH-pretreatment and 43.1% higher than tests with untreated corn stover. This increase was attributed to the synergistic effect from NaOH pretreatment and trace elements addition. The synergistic effect was mainly due to the complementary characteristics of digested corn stover, more balanced nutrients, and improved biodegradability.

Multiple comparisons using the LSD method were performed on the cumulative biogas productions for the three groups. Test results showed that the cumulative biogas production with the A1B3C2 group was significantly higher than those of the others (*a* = 0.05). It further confirmed that combining NaOH pretreatment with trace elements could significantly improve biogas production when corn stover was used as a sole feedstock.

The energy contained in biogas is determined using both biogas volume and BVF. Cumulative biomethane volume, representing total energy gain, was calculated by timing daily biogas production with the corresponding BVF.  Figure 2(b) shows the results. When anaerobic fermentation processes were completed, the cumulative biomethane volume reached 11,367 mL, 8,018 mL, and 7,009 mL for the A1B3C2, NaOH-pretreated, and untreated corn stover groups, respectively. The A1B3C2 group's cumulative biomethane volume was 41.8% higher than the NaOH-pretreated group and 62.2% higher than the untreated corn stover group, respectively. This confirms the significant influence of trace elements and NaOH pretreatment on biomethane production. Multiple comparisons using the LSD method also showed a significant difference (*a* = 0.05) in cumulative biomethane volume among three groups. Speece also demonstrated that adding trace elements of Fe, Co, and Ni could significantly increase biomethane production [16].

#### 3.2.3. Digestion Time T_90_

Digestion time is another indicator of substrate biodegradability and digestion efficiency. Digestion time T_90_ was defined as the number of days required to achieve 90% of potential biogas generation.

This study's anaerobic digestion process was extended for up to 50 days past the time when biogas production was near zero. The T_90_ for the A1B3C2 group was 33 days, 5–9 days shorter than NaOH-pretreated and untreated groups. The significant reduction in digestion time further indicated that the A1B3C2 group not only initiated digestion quickly, but also accelerated the biogas production process. Gonzalez-Gil et al. [17] studies indicated that adding Ni and Co can shorten the reaction's lag phase and facilitate the methanogen process [17]. This could bring significant economic benefits, by increasing the production efficiency or treatment capacity of a digester by using a shortened digestion time.

### 3.3. Mass Bioconversion

#### 3.3.1. Bioconversion of TS and VS

Biogas is generated from a substrate's biological conversion during anaerobic digestion. Organic matter conversion into biogas reduces the amount of organic dry matter, resulting in a decrease in TS and VS.

Based on mass balance, the TS and VS bioconversion rates were calculated; Table 5 shows the results. Both TS and VS were reduced significantly through anaerobic microorganism bioconversion. However, the TS and VS bioconversion rates differed from each other and also differed between the three groups. The TS bioconversion rate was lower than VS for all three groups. TS and VS bioconversion rates for the A1B3C2 group were 55.7% (TS) and 65.9% (VS). These rates were 22.2%–56.3% higher than with the NaOH-pretreated group and 14.0%–53.1% higher than with the untreated corn stover. The A1B3C2 group achieved a higher bioconversion rate, representing significant biodegradability improvement. This is attributed to the combined role of NaOH pretreatment and trace elements, which improved corn stover biodegradability and also provided more balanced nutrients for anaerobic bacteria.

#### 3.3.2. Bioconversion of Chemical Compositions

LCH (lignin, cellulose, and hemicellulose) are the main components of corn stover, accounting for 75.0% of the total dry matter and providing the main carbon sources for anaerobic microorganisms. Biogas production is greatly affected by the availability and digestibility of cellulose and hemicellulose and the association of lignin with the carbohydrates. The more biogas produced, the more the components are reduced. In this part of the study, cellulose, hemicellulose, and lignin bioconversion rates were analyzed to investigate main component bioconversion characteristics.

**Figure 3:**
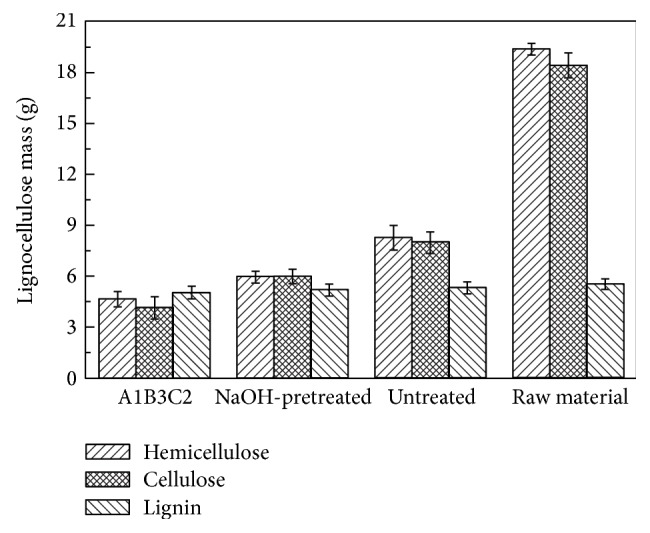
The changes of chemical compositions.

Figure 3 shows the changes in chemical compositions for the three studied groups at the end of anaerobic digestion. Bioconversion rates of the cellulose, hemicellulose, and lignin differed across the three groups. Hemicellulose and cellulose were clearly converted for all three groups. The total LCH bioconversion rates were 72.8%, 65.1%, and 54.9% for the A1B3C2, NaOH-pretreated, and untreated groups, respectively, when compared to raw material. The bioconversion rates of hemicellulose significantly increased by 76.1%, 69.3%, and 57.5% for the A1B3C2, NaOH-pretreated, and untreated groups, respectively. The bioconversion rates of cellulose significantly increased by 77.5%, 67.5%, and 56.7% for the A1B3C2, NaOH-pretreated, and untreated groups, respectively. Lignin amounts had almost no change. The A1B3C2 group achieved the maximum LCH bioconversion rate.

Pretreatment before anaerobic digestion is a simple and effective method to improve lignocellulosic material biodegradability. Pretreatment can decompose cellulose and hemicellulose into relatively biodegradable components and break the link between polysaccharide and lignin to make cellulose and hemicellulose more accessible to bacteria [18]. Additionally, adding trace elements for corn stover anaerobic digestion can enhance microorganism activity. This result further verified NaOH pretreatment effectiveness and the ability of trace elements to improve biodegradability and enhance bioenergy production.

#### 3.3.3. Trace Element Bioavailability

Methanogens absorbed and fixed trace elements through extracellular complexion, extracellular precipitation, and intracellular accumulation [19]. Fe, Co, and Ni concentrations in effluent were measured to assess trace element availability at the end of anaerobic digestion.  Table 6 lists the trace elements present in the feedstock and effluent from the A1B3C2 group. The table shows that effluent trace elements mainly existed as solids in the A1B3C2 group; the amounts of trace elements in the solids accounted for 93.8%, 96.1%, and 91.1% of total Fe, Co, and Ni amounts added, respectively. Karlsson et al. (2012) also noted that only a fraction of trace elements is present in solution; in most cases, trace element bioavailability for anaerobic bacteria metabolic pathways is not related to the total amount in the medium [11]. The calculated result shows that approximately 89.6% Fe, 97.2% Co, and 87.4% Ni of the total amount added were converted to solid form. As such, adding Fe 0.897 g, Co 0.389 g, and Ni 0.349 g at the beginning of operation can both satisfy anaerobic digestion needs and enhance biological activity.

### 3.4. System Stability

It is important that a digester operate in a stable state, while maintaining good performance. When using corn stover as a sole substrate, the digestion system has an increased potential for instability, due to possible lower buffering capability. Digestion system stability depends on a number of factors, including pH, volatile fatty acids (VFAs), ammonia nitrogen, and alkalinity. This study assessed these parameters to evaluate the stability of corn stover anaerobic digestion when combining NaOH pretreatment and trace element supplements.

Table 7 shows that each group's pH value was maintained at 7.30–7.45 at the end of methanogenesis throughout the digestion period. Ammonia nitrogen and alkalinity were important parameters in maintaining anaerobic system stability. Free ammonia was produced from organic nitrogen degradation, causing alkalinity variation in the anaerobic system. Adequate ammonia content can effectively improve the efficiency of anaerobic digestion, while ammonia nitrogen may inhibit methanogen activity when levels exceed 2,000 mg/L. Table 7 shows that the ammonia nitrogen for the three groups ranged from 490 to 518 mg/L, within an acceptable range. The anaerobic digestion system alkalinity was 8,550–10,700 mg/L. This higher alkalinity supports strong system stability. These findings indicate that the corn stover with NaOH pretreatment and trace element supplements did not inhibit ammonia nitrogen, while maintaining high alkalinity, thereby ensuring stable system operation.

VFAs were important intermediate products during anaerobic digestion. VFAs in the A1B3C2 group were significantly lower than those with NaOH pretreatment and untreated group (Table 6). The A1B3C2 group benefited methanogenic bacterial growth and biomethane production.

## 4. Conclusions

Combining NaOH pretreatment and trace element addition is an effective method to improve corn stover biodegradability and enhance biomethane production. The best combination was adding 1.0, 0.4, and 0.4 mg·L^−1^·d^−1^ of trace elements Fe, Co, and Ni (A1B3C2), respectively. When compared to NaOH-pretreated and untreated corn stover, A1B3C2 group experienced 41.8% and 62.2% more cumulative biomethane volumes, 22.2%–56.3% and 14.0%–53.1% more TS and VS bioconversion rates, and 5–9 days shorter T_90_, while also maintaining good operational stability.

## Figures and Tables

**Table 1:** Characteristics of corn stover and activated sludge used in this study.

Indexes (dry matter)	Corn stover	Activated sludge
Total solid (%)	94.93 ± 0.36	14.33 ± 0.13
Volatile solid (%)	85.14 ± 0.24	5.95 ± 0.19
Total carbon (%)	42.65 ± 0.14	30.12 ± 0.39
Total nitrogen (%)	1.22 ± 0.17	3.28 ± 0.32
Cellulose (%)	38.82 ± 0.43	—
Hemicellulose (%)	29.02 ± 0.37	—
Lignin (%)	7.16 ± 0.14	—
Fe (mg/Kg)	624.59 ± 12.85	8559.60 ± 78.43
Co (mg/Kg)	0.39 ± 0.01	3.55 ± 0.01
Ni (mg/Kg)	7.45 ± 0.12	14.81 ± 0.39

**Table 2:** Factors and levels of coding table.

Factor	Level			
	1 mg·L^−1^·d^−1^	2 mg·L^−1^·d^−1^	3 mg·L^−1^·d^−1^	Compounds
A (Fe)	1.00	5.00	10.00	FeCl_2_·4H_2_O
B (Co)	0.05	0.20	0.40	CoCl_2_·6H_2_O
C (Ni)	0.20	0.40	0.60	NiCl_2_·6H_2_O

**Table 3:** Analysis of full-factor test.

Experimental group	Codes of factor and level (actual levels)			Cumulative biomethane production (mL)
	A (Fe) mg·L^−1^·d^−1^	B (Co) mg·L^−1^·d^−1^	C (Ni) mg·L^−1^·d^−1^	
1	1 1.00	1 0.05	1 0.20	10386 ± 94
2	1 1.00	1 0.05	2 0.40	10001 ± 56
3	1 1.00	1 0.05	3 0.60	10666 ± 396
4	1 1.00	2 0.20	1 0.20	10730 ± 62
5	1 1.00	2 0.20	2 0.40	10323 ± 491
6	1 1.00	2 0.20	3 0.60	9638 ± 365
7	1 1.00	3 0.40	1 0.20	10320 ± 118
8	1 1.00	3 0.40	2 0.40	11367 ± 361
9	1 1.00	3 0.40	3 0.60	11132 ± 163
10	2 5.00	1 0.05	1 0.20	9593 ± 274
11	2 5.00	1 0.05	2 0.40	10517 ± 141
12	2 5.00	1 0.05	3 0.60	10839 ± 239
13	2 5.00	2 0.20	1 0.20	10322 ± 456
14	2 5.00	2 0.20	2 0.40	9868 ± 259
15	2 5.00	2 0.20	3 0.60	10076 ± 388
16	2 5.00	3 0.40	1 0.20	10307 ± 101
17	2 5.00	3 0.40	2 0.40	10158 ± 560
18	2 5.00	3 0.40	3 0.60	10861 ± 76
19	3 10.00	1 0.05	1 0.05	10274 ± 367
20	3 10.00	1 0.05	2 0.40	10504 ± 649
21	3 10.00	1 0.05	3 0.20	10340 ± 103
22	3 10.00	2 0.20	1 0.05	10127 ± 441
23	3 10.00	2 0.20	2 0.40	10678 ± 582
24	3 10.00	2 0.20	3 0.20	10116 ± 33
25	3 10.00	3 0.40	1 0.05	10872 ± 156
26	3 10.00	3 0.40	2 0.40	10767 ± 372
27	3 10.00	3 0.40	3 0.20	9837 ± 105

**Table 4:** The result of between-subjects effects (dependent variables: *X*).

Source	III model 0	df	Mean square	*F*	Sig.

Correcting model	42544349.6^a^	26	1636321.137	2.635	0.001
Intercept	22460617248	1	22460617248	36165.606	0.000
A	933550.222	2	466775.111	0.752	0.476
B	6618446.889	2	3309223.444	5.328	0.008
C	644693.556	2	322346.778	0.519	0.598
A ∗ B	2944740.444	4	736185.111	1.185	0.328
A ∗ C	6475833.778	4	1618958.444	2.607	0.046
B ∗ C	8525127.111	4	2131281.778	3.432	0.014
A ∗ B ∗ C	16401957.556	8	2050244.694	3.301	0.004

Error	33536652.000	54	621049.111		

Total	22536698250.000	81			

Total correction	76081001.556	80			

^a^ *R* ^2^ = 0.559 (regulate *R* ^2^ = 0.347).

**Table 5:** Biogas and biomethane yields and bioconversion rates for different groups.

Groups	Biogas yields		Biomethane yields		Bioconversion rates (%)	
	mL/g TS	mL/g VS	mL/g TS	mL/g VS	TS	VS
A1B3C2	354 ± 17	393 ± 31	219 ± 12	243 ± 24	55.7 ± 0.5%	65.9 ± 0.6%
NaOH-pretreated	277 ± 23	307 ± 26	154 ± 16	171 ± 20	45.6 ± 0.2%	57.8 ± 0.5%
Untreated	247 ± 15	275 ± 18	135 ± 14	150 ± 19	35.6 ± 0.9%	53.0 ± 0.3%

**Table 6:** The amount of trace elements in feedstock and effluent.

Element	Feedstock (mg)			Effluent (mg)	
	Activated sludge	Corn stover	Element addition	Liquid	Solid
Fe	122.16 ± 0.23	32.47 ± 0.85	48.00	12.66 ± 0.21	192.18 ± 1.42
Co	0.19 ± 0.01	0.07 ± 0.01	19.47	0.78 ± 0.01	19.20 ± 0.12
Ni	0.21 ± 0.01	0.39 ± 0.02	19.20	1.70 ± 0.13	17.38 ± 0.16

**Table 7:** System stability of anaerobic digestion.

Different groups	pH	Ammonia nitrogen (mg/L)	Alkalinity (mg/L)	Volatile fatty acids (mg/L)
A1B3C2	7.44 ± 0.02	490 ± 30	8550 ± 850	269 ± 50
NaOH-pretreated	7.45 ± 0.02	518 ± 56	10700 ± 300	511 ± 10
Untreated	7.47 ± 0.02	490 ± 42	9255 ± 225	785 ± 21
